# Fluoride Exposure Increases the Activity of the Cystine/Glutamate Exchanger in Glia Cells

**DOI:** 10.1007/s11064-025-04358-2

**Published:** 2025-02-25

**Authors:** Andrea Ocharán-Mercado, Jaqueline Loaeza-Loaeza, Daniel Hernández-Sotelo, Luis Cid, Luisa C. Hernández-Kelly, Marie-Paule Felder-Shmittbuhl, Arturo Ortega

**Affiliations:** 1 Departamento de Toxicología, Centro de Investigación y de Estudios Avanzados del Instituto Politécnico Nacional, Av. IPN 2508, San Pedro, Zacatenco, 07360 México; 2 Laboratorio de Epigenética del Cáncer, Facultad de Ciencias Químico-Biológicas, Universidad Autónoma de Guerrero, Av. Lázaro Cárdenas 88, 39086 Chilpancingo, Guerrero México; 3 Centre National de La Recherche Scientifique, Institut Des Neurosciences Cellulaires Et Intégratives (UPR 3212), Université de Strasbourg, Strasbourg, France

**Keywords:** Fluoride, Glutamate/cystine exchanger, HuR, Glia

## Abstract

Fluoride exposure in drinking water has been widely related to impairment of cognitive function. Even though this ion has been described as neurotoxic for more than two decades, the molecular mechanisms of fluoride neurotoxicity are not fully understood, however, increasing evidence suggests that glial cells are the site of early injury in fluoride neurotoxicity. Nevertheless, a convergence point of many studies is the effect on glutamatergic neurotransmission and the generation of reactive oxygen species. In this context, we evaluated here the expression and regulation of the cystine/glutamate exchanger upon fluoride exposure since this transporter is in the interface between excitotoxicity and the antioxidant response. We demonstrate here the functional expression of the cystine /glutamate exchanger in both the U373 human glioblastoma cells and chick cerebellar Bergmann glia cells. Using a [^3^H]-L-Glutamate uptake assay, we demonstrate that fluoride increases the activity of the exchanger in a time and dose-dependent manner. This augmentation is mitigated by the antioxidant Trolox. To gain insight into fluoride neurotoxicity mechanisms, we evaluated its effect on human antigen R, a RNA binding protein, that binds to the 3'-UTR region of exchanger mRNA increasing its half time life. An increase in human antigen R protein was recorded after a 6 h fluoride exposure, suggesting that this ion regulates the exchanger through this RNA-binding protein. Furthermore, we show that fluoride exposure increases both the exchanger and human antigen R mRNAs half-life. These results provide insights into fluoride neurotoxicity mechanisms and support the notion of a central role of glial cells in neuronal glutamatergic transmission disruption that leads to neuronal cell death.

## Introduction

Fluoride (F^−^), an ion originating from fluorine, is broadly found in soil, and released into the environment mainly through the dissolution of minerals deposited in the subsoil and groundwater [1] and can come into contact with humans. As defined by the World Health Organization (WHO), water supplementation with F^−^ (0.5 to 1.0 mg/L) enhances dental health [2] while chronic ingestion of over 1.5 mg/L F^−^ can result in toxic effects in multiple organs including the brain [3, 4]. It causes neurodevelopmental alterations both in animals and humans that lead to a sharp decrease in the intelligence quotient (IQ) and impair learning and memory processes [5, 6]. Global and national regulations establish 1.5 mg/L as the permissible F^−^ limit for water for human consumption [7], however in some locations, F^−^ levels can reach up to 23.4 mg/L [8]. Although the study of F^−^ neurotoxicity begun in the last century, it was not until recently that this ion was defined as a neurotoxicant [9].

Research on F^−^ metabolism confirmed that it can cross the blood-placental barrier (BPB) [10, 11]. Mice exposed to 5–50 mg/L of NaF^−^ in drinking water for 2–12 weeks showed increased plasma F^−^ bioavailability and concentrations around 200 mg/kg brain tissue, adversely affecting the development of neurons and glial cells of the Central Nervous System (CNS) altering brain maturation and triggering of short- and long-term memory impairments [10, 12–14]. It is important to mention that rodent models show resistance to F^−^ toxicity of 5–tenfold compared to humans, pointing out that 50 mg/L is equivalent to 10 mg/L or 500 µM in humans (concentration used in this study), which has been described as endemic in several regions in Mexico and worldwide [10, 13, 15–18].

As a result of the high amount of polyunsaturated fatty acids and low levels of antioxidant enzymes, the brain is susceptible to oxidative stress [19], this phenomenon can be over-regulated by free radical-inducing agents, such as F^−^, this ion upregulates ROS production leading to oxidative damage which could be a factor that causes the impairment of glutamatergic transmission [3]. It has been demonstrated that Glutamate (Glu)-mediated signaling is involved in the regulation of cognitive processes, therefore, any alteration of the proteins involved in the Glu/glutamine shuttle modifies neurotransmission and consequently learning processes. Glu is the main excitatory neurotransmitter in the CNS and participates in processes related to motor activity, learning, and neuronal plasticity [20, 21]. Due to the lack of Glu degrading enzymes in the synaptic cleft, clearance systems, present mainly in glial cells, are needed to prevent Glu receptors over-stimulation and neurotransmitter spillover [22, 23]. Glu levels in the synaptic cleft are mainly regulated through excitatory amino acid transporters (EAAT) 1 and 2 and the system xc- [24, 25].

System xc- is a heteromeric plasma membrane amino acid transporter consisting of two subunits: a lower molecular weight light chain called xCT, which confers the specificity of substrate transport (cystine/Glu) and a higher molecular weight heavy chain, named 4F2hc [26]. xCT, a protein encoded by the *slc7a11* gene has several AU-rich elements (AREs) [27]. The exchanger transport stoichiometry consists of internalizing one molecule of cystine (Cys) and exporting one molecule of Glu in a Na^+^-independent manner, the transport direction can be reversed by the substrate concentration [28]. Internalized cystine functions as a precursor of glutathione (GSH), the most important endogenous antioxidant in the CNS, therefore xCT plays an important role in maintaining intracellular redox balance and is also involved in extracellular Glu levels [29]. Under conditions of oxidative stress, such as F^−^ exposure, EAATs and xCT are required for GSH synthesis and which is limited by cystine availability. Nevertheless, under oxidative stress, a significant release of Glu occurs associated with Cys input through the xCT exchanger, resulting in a sustained synaptic and extra-synaptic Glu receptors that leads to neuronal and oligodendrocyte death in a phenomena known as excitotoxicity [30]. Both endogenous and exogenous compounds can induce xCT up-regulation in the plasma membrane [31–33].

In recent years, accumulating evidence supports the involvement of RNA-binding proteins (RBPs) in post-transcriptional gene expression regulation [27]. These proteins are regarded as RNA-binding proteins [34], able to bind mRNA. Among their nuclear functions are regulating alternative splicing and mRNA export, whereas among their cytoplasmic functions are regulating mRNA stability and translation, thereby fine-tuning protein levels. [35]. Particularly, human antigen R (HuR), an RBP protein encoded by the ELALVL1 gene, is well characterized to bind AREs transcripts [36] and might play a pivotal role in xCT mRNA stability through its binding to xCT 3'-UTR end, augmenting its stability, and protecting it from rapid degradation by exonucleases and by these means increasing the transporters’ translation [37]. Although HuR has been traditionally related to tumor progression, it has recently been shown to be associated with neurodegenerative diseases such as sensory neuropathy, multiple sclerosis, spinal muscular atrophy, amyotrophic lateral sclerosis, glioma, and paraneoplastic encephalomyelitis among others [37–39].

Astrocytes outnumber neurons in several brain structures; these cells completely envelop excitatory and inhibitory synapses [40] and participate in the *so called* Glu/glutamine shuttle that is the biochemical firm of Glu turnover [41]. Although xCT and HuR are expressed in healthy astrocytes, their expression is higher in cancer cells [42]. In this context, our main goal was to study the expression of these proteins in response to F^−^ exposure, that as noted above, results in reactive oxygen species (ROS) production in a similar scenario as in transformed cells. To this end, we used the U373 astrocytoma cell line in which these proteins are overexpressed. To confirm our results, we also used a well-characterized glial primary culture, the chick cerebellar Bergmann glia cells (BGC) culture [43]. To gain insight into the molecular mechanisms of F^−^ neurotoxicity and its clear target to glutamatergic transmission, we explored a plausible pathway for xCT dysregulation function due to exposure to this contaminant. xCT mRNA has a long 3'-UTR that can act as a binding site for RNA-binding proteins such as HuR. With this in mind, we decided to evaluate whether F^−^ modified the activity of cystine/glutamate exchanger and gain insight into its plausible involvement in the regulation of the exchanger.

## Methods

### Materials

The human glioblastoma cell line (U373-MG) was obtained from the American Type Culture Collection (Manassas, VA, USA). Dulbecco’s modified Eagle’s Medium F-12 (DMEM-F12) and Opti-MEM were obtained from Gibco BRL (Gaithersburg, MD, USA). Fetal bovine serum was bought from PAN BIOTECH (Germany). Antibiotic was obtained from AAS-B Capricorn Scientific (Ebsdorfergrund, Germany). Dimethyl POPOP and 2,5-Diphenylozazole-scintillation grade were obtained from Research Products International (Mount Prospect, IL, US). L-Quisqualic acid and Trolox were purchased from Tocris Bioscience (St. Louis, MO, USA). Anti HuR monoclonal antibodies were obtained from Santa Cruz (CA, USA; sc-5261). Anti-xCT antibodies were purchased from Abcam (Cambridge, UK; ab37185). Anti-mouse IgGs were obtained from Jackson Immuno Research (Cambridge, UK). Alexa 488 was obtained from Thermo Fisher (Massachusetts, USA). Bradford and Acrylamide were obtained from BioRad (Hercules, CA, USA). N–N´ Methylenbisacrylamide was purchased from Bethesda Research Laboratories (Bethesda, MD, USA). CYBRFast 1-step RT-qPCR Lo- ROX Kit was obtained from Tonbo Biosciences (San Diego, CA, USA). Trizol was obtained from Invitrogen (Waltham, MA, USA). L-[3,4-^3^H]-Glutamic acid, specific activity 40 Ci/mmol was purchased from ARC (St. Louis, MO, USA). Plasticware was purchased from Corning (New York, NY, USA). Sodium fluoride, cytochalasin B, L-Glutamic acid potassium salt monohydrate, Fluoroshield, DAPI, Ponceau, MTT and all other chemicals were obtained from Sigma-Aldrich (St. Louis, MO, USA).

### Cell Culture and Fluoride Stimulation Protocol

Primary cultures of Bergmann glia cells (BGC) were obtained after minor modifications from our previously described protocol [43]. Cells were seeded in Opti-MEM supplemented with 2.5% fetal bovine serum (FBS), and 1% antibiotic solution gentamicin under standard conditions (37 °C, 5% CO_2_ and 95% humidity). U373-MG cells were cultured in Gibco Dulbecco's Modified Eagle Medium: Nutrient Mixture F12 (DMEM-F12) containing 10% FBS and 1% of the antibiotic solution. Cells were incubated under standard conditions. For reseeding, the culture medium was removed, and monolayers were washed with phosphate buffer solution (PBS), cells were detached with PBS/EDTA by incubating for 5 min at room temperature, once detached, the medium was added to stop the reaction and then low-speed centrifugation was used to isolate the cells. The cells were then seeded in 6, 24, or 96-well microplates and used in the experiments at 85% confluence. Uptake assay treatments were carried out in an assay buffer solution without sodium (25 mM HEPES-Tris, 130 mM choline chloride, 5.4 mM KCl, 1.8 mM CaCl_2_, 0.8 mM MgCl_2_, 33.3 mM glucose, and 1 mM K_2_HPO_4_, pH = 7.4).

### Cell Viability Assays

To perform these experiments, U373-MG cells were plated in 96-well culture plates until reaching 70% confluence. The viability was measured by two different methods, first it was evaluated by the 3-(4,5-dimethylthiazol-2-yl)−2,5-diphenyltetrazolium bromide assay (MTT), which determines the ability of metabolically active cells to produce a purple formazan salt after the cleavage of the tetrazolium ring of a yellow MTT substrate by mitochondrial reduction [44]. The amount of formazan was determined at λ = 570 nm and it is directly proportional to the number of viable cells. Cells were treated with different F^−^ concentrations for 24 h (10, 50, 100, 200, 500, 1000 µM), and 1% Triton X-100 was used as a positive control of cell death. Three hours before the treatment ended, 20 µL/ml of an MTT stock solution (0.5 mg/ml) was added directly into each well, and the plates were returned to the incubator. Finally, the medium was discarded, and 180 µL of DMSO was added to each well to dissolve the formazan crystals. Absorbance was measured with a microplate reader (Epoch, BioTek Instruments, VT, USA).

F^−^ cytotoxicity was also determined by the neutral red assay (NR) which consists on the uptake and subsequent lysosomal accumulation of the supravital dye, NR [45]. Quantitation of the dye extracted from the cells is linearly correlated to the number of living cells, both by direct counts and by protein determination [46]. Cells were treated with different F^−^ concentrations (10, 50, 100, 200, 500, 1000 µM), and 1% Triton X-100 was used as a positive control of cell death. After exposure, the medium was discarded; and the cells were washed twice with PBS per well, then an aliquot the NR-containing medium (pre-incubated overnight at 37 °C and centrifuged prior to use to remove fine precipitates of dye crystals) was added to each well. The plates were incubated for 2 h room temperature to allow the uptake of the dye into the lysosomes of viable cells. After that, the neutral red medium was removed; the cells were washed with PBS and NR detainer solution (50% ethanol, 49% deionized water, 1% glacial acetic acid) was added, and the plate was shaken rapidly on a microtiter plate until a homogenous solution was reached the plate was transferred to a plate reader at λ=570 nm and the absorbance was measured with a microplate reader (EPOCH, BioTek Instruments, VT, USA).

### Staining Procedures

BGC and U373 cells were seeded on glass coverslips, exposed for 30 min to 500 µM F^−^ and fixed at − 20 °C with methanol. Cells were rinsed with PBS. Non-specific binding was prevented by incubation with blocking solution (1% BSA, 0.01% Triton X-100 in PBS) for 2 h. Coverslips were rinsed twice with PBS. Cells were exposed to a 1:300 dilution of the primary antibody anti-xCT, in blocking solution overnight at 4 °C, followed by the incubation with fluorescein-labeled goat anti-rabbit (1:1000) antibodies for 2 h at room temperature. Preparations were mounted with Fluoroshield/DAPI. Cell preparations were examined under confocal microscopy (Nikon ECLIPSE Ti Series, Inverted Microscope Systems). Pictures were processed with the FIJI software.

### [^3^H]-L-Glutamate Uptake

Confluent U373-MG monolayers seeded in 24-well plates were pre-incubated with F^−^, H_2_O_2_, cytochalasin B, inhibitors, and competitors in assay buffer without sodium at the indicated concentrations and periods. Uptake experiments were made with a fixed 0.4 mCi/ml [^3^H]-L-Glu amount, and the indicated L-Glu concentrations by the method of isotope dilution [47]. For one-point assays the L-Glu final concentration was 25 µM and the uptake time for 30 min. The uptake was halted by rapid aspiration of the radiolabeled medium and each well was rinsed with ice-cold assay buffer in a 15 s interval. The monolayers were solubilized with 0.1 M NaOH for 2 h at room temperature, an aliquot was used for protein determination by the Bradford method. The radioactivity associated with the solubilized suspension was determined in a Perkin Elmer scintillation counter. A minimum of three independent experiments in quadruplicates were carried out. To calculate the kinetic constants, curves were analyzed by Michaelis–Menten saturation assays, and for IC_50_ calculation, data was analyzed by log (inhibitor) vs response (nonlinear regression) using GraphPad Prism Software (CA, USA).

### SDS-PAGE and Western Blots

Cells from confluent monolayers were harvested with PBS containing phosphatase inhibitors (10 mM NaF, 1 mM Na_2_MoO_4_ and 1 mM Na_3_VO_4_). The cells were lysed with 50 mM Tris–acetate, 5 mM EDTA, and 1 mM PMSF. Approximately 100 µg of protein, as determined by the Braford method, were denaturized in Laemmli's sample buffer, proteins were resolved through a 10% SDS-PAGE and then transferred to nitrocellulose membranes. Membranes were stained with Ponceau Red to confirm that proteins were correctly transferred and equal protein concentration present in all lanes. Membranes were soaked in PBS to remove the stain and incubated in TBS containing 5% dried skimmed milk and 0.1% Tween 20 for 120 min to block the excess of non-specific protein binding sites. Membranes were then incubated overnight at 4 °C with primary antibodies, followed by secondary anti-mouse IgGs for 2 h at room temperature. Immunoreactive polypeptides were detected by chemiluminescence and exposed to X-ray films. Densitometry analyses were performed, and data was analyzed with Image J software.

### RNA extraction and qRT-PCR

Confluent monolayers were pretreated with F^−^ for different time periods to evaluate *slc7a11* and *elalvl1* mRNAs. Total RNA was isolated from confluent U373-MG cell cultures and extracted using TRIZOL. PCR was performed in a reaction volume of 10 μL. Quantitative real-time reverse transcription-PCR (qRT-PCR) was performed by a one-step method with 100 ng of total RNA using CYBRFast 1-step RT-qPCR Lo-ROX Kit. Samples were subjected to quantitative PCR (qPCR) using Step One Plus Real-time PCR System (Applied Biosystems). The qPCR profile consisted of an initial cDNA synthesis by Reverse Transcriptase at 50 °C for 10 min, an inactivation of the reverse transcriptase at 95 °C for 2 min, followed by 40 cycles of 95 °C for 10 s, 60 °C or 68 °C per 30 s. To quantify SLC7A11 and HuR mRNA levels we standardized the amplification temperature, ELALVL1 Forward 5′-CGCCAACTTGTACATCAGCG-3′ and ELALVL1 Reverse 5′-TAAACGCAACCCCTCTGGAC3′. SLC7A11 Forward 5′-CATGAGTGTCAGCTGGAGCGCC-3′ and SLC7A11 Reverse 5′-GCCAGTGGCAACCGCGTAATAC-3′. As an endogenous control, we used GAPDH designed by Merck. The conditions for qRT-PCR were standardized. The relative abundance of HuR and xCT mRNA is expressed as a sample versus a control normalized to GAPDH mRNA levels and was calculated as 2^−ΔΔCT^. To determine whether F^−^ altered xCT mRNA stability, astrocytes were treated with actinomycin D (ActD; 8 µg/ml) added to stop transcription. At different times following the addition of ActD, RNA was isolated, and then HuR and xCT mRNA levels were determined using RT-qPCR (see above). To calculate mRNA half-life, curves were analyzed using one-phase exponential decay using GraphPad Prism.

### Statistical Analysis

Data are presented as the mean ± SEM from at least three independent experiments. One-way or two-way ANOVA analysis of variance was carried out to determine significant differences between conditions followed by Dunnett’s multiple comparison or Tukey test, according to the results. Differences with a p ≤ 0.05 value were considered statistically significant. All the plots and analyses were performed with GraphPad Prism 8.0 Software (CA, USA).

## Results

### Fluoride Does not Exhibit Cytotoxic Effects in Astrocytes

**Fig. 1:**
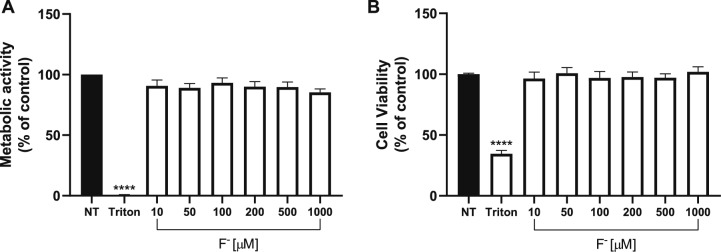
F^−^ has no cytotoxic *effects on U373-MG cells.* **A** Metabolic activity (MTT assay) and **B** viability (Neutral red) in U373-MG glial cells exposed to F^−^. Cells were treated with 10% Triton X-100 as death control and increasing fluoride concentrations for 24 h. Data are expressed as mean ± SEM of 3 independent experiments in quadruplicate (n = 3). Each bar was compared vs the non-treated group and analyzed with one-way ANOVA followed by Dunnett's test post-hoc test. ****p < 0.0001 vs control

Chronic F^−^ exposure can result in toxic effects such as *fluorosis* and a decrease in intelligence quotient (IQ) leading to disturbances in learning and memory in humans and animals [14]. It has been reported that F^−^ can cause cytotoxic effects, mostly mediated by the inhibition of enzymes that use Mg^2+^ is a co-factor, which would potentially lead a metabolic disturbances and even cell death [48]. Therefore, we first decided to evaluate cell viability under F^−^ exposure, the results are presented in Fig. 1A and B, a 24 h F^−^ exposure in a concentration range of 10 to 1000 µM is insufficient to produce a significant diminution in cellular viability as measured by both MTT (Panel A) and Neutral Red (Panel B), demonstrating that U373-MG cells are resilient to the cytotoxic effects of F^−^, even at the maximum concentration (1000 µM). As expected, exposure to 1% Triton X-100 reduced cell viability. Regarding the viability test in BGC, Flores-Méndez previously determined that 500 M F^−^ does not produce a significant decrease in viability [49].

### xCT is Expressed in Bergmann Glial and U373 Cells

**Fig. 2:**
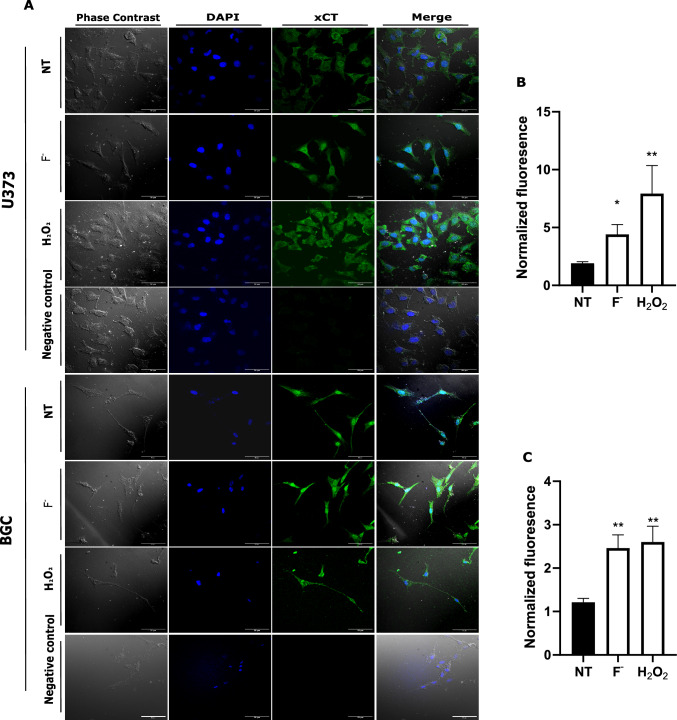
xCT immunoreactivity *in U373 and BGC cultures.* **A** U373 and BGC cells were exposed to 500 µM F^−^ for 30 min (F^−^), or 0.3 mM H_2_O_2_ for 10 min. Fixed cells were incubated 12 h with anti-xCT antibodies. A negative control (no primary antibodies) is shown. Phase contrast (gray), DAPI counter-stained nucleus (blue), xCT (green), and merge images are shown. Scale bar 50 µm. In panels **B** and **C** U373 and BGC respectively normalized fluorescence is expressed, NT cells are taken as 1. A representative image of 3 independent experiments of each cell line is shown. Statistical analysis was done by a one-way ANOVA followed by Dunnett’s multiple comparison test. Results are the mean ± SEM of 3 independent experiments (n = 3). *p < 0.05, **p < 0.001, vs control. The exposure time and conditions during the acquisition of photographs and the gain were always the same under the different conditions

Given the well-known role of xCT in extracellular Glu maintenance and cystine uptake, we explored the expression of this exchanger in our models after F^−^ and H_2_O_2_ exposure. To this end, an immunocytochemical approach was used. The results are depicted in Fig. 2, in both U373 and BGC cultures, xCT immunoreactivity is present in control conditions (NT), interestingly, after a 30 min 500 µM F^−^ the xCT signal is increased by 400% in U373-MG cells (Fig. 2B) and 200% (Fig. 2C) in BGC. As expected, a 0.3 mM H_2_O_2_ treatment reproduces this effect suggesting a ROS-mediated effect [50]. We are aware of the limitation of commercially available xCT antibodies specificity [25], therefore, these increases can overrepresent visualized results in immunofluorescence, however, truly F^−^ stimulus can overexpress xCT in our models.

### F^−^ Increases Cystine/Glutamate Exchanger Mediated Glu Uptake

**Fig. 3:**
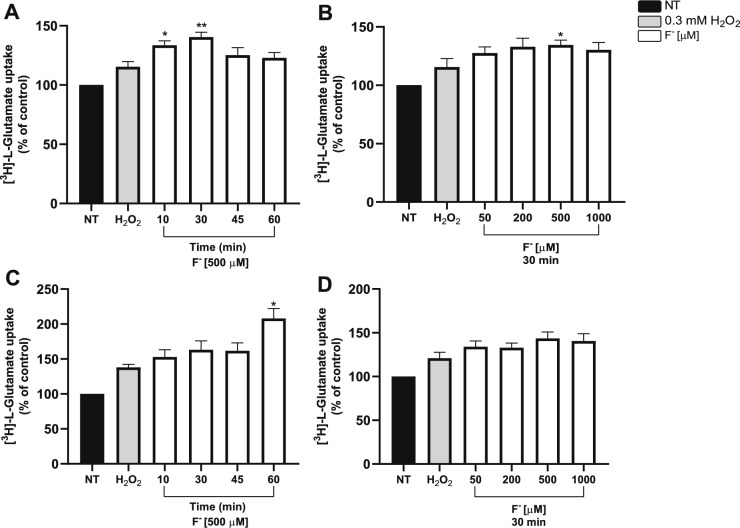
Characterization *of cystine/glutamate exchanger activity upon F*^−^ exposure. **A** U373 cells were exposed to 500 µM F^−^ for 10, 30, 45, and 60 min. **B** U373 cells treated with 50, 200, 500 and 1000 F^−^ µM for 30 min. **C** BCG exposed to 500 µM F^−^ for 10, 30, 45, and 60 min. **D** BGC treated with 50, 200, 500 and 1000 F^−^ µM for 30 non-treated cells (NT) were used as a control; and 0.3 mM H_2_O_2_ was used as a positive control. Each bar was compared to the control. Statistical analysis was performed by one-way ANOVA followed by Dunnett’s multiple comparison test. Results are the mean ± SEM of 3 independent experiments; each experiment was performed in quadruplicates (n = 3). *p < 0.05, **p < 0.01, vs control

In the CNS, cystine/glutamate exchanger is expressed in neurons [51], microglia [52], and astrocytes [25]. Interestingly, glioma cells express a higher amount of system xc- compared to astrocytes [53]. With this in mind, we used the U373-MG cell line together with the well characterized chick cerebellar BGC culture to explore if xCT might be a molecular target of F^−^. Glioma cells were pre-treated with F^−^ for different time periods and concentrations, and the cystine/glutamate exchanger activity evaluated through [^3^H]-L-Glu uptake assays, based on an isotope dilution approach [47]. We performed uptake assays in sodium-free conditions, to avoid excitatory amino acid transporters (EAATs) activity. A final 25 µM Glu concentration was used to match with the system xc- affinity constant reported by our group in cerebellar BGC [31]. A time and dose dependent increase in Glu uptake was present after F^−^ pre-treatment (Fig. 3).

### Cystine/Glutamate Exchanger Specificity and its Regulation by F^−^

**Fig. 4:**
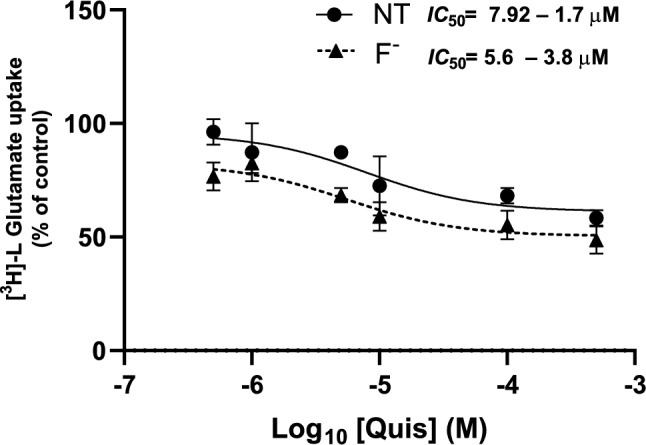
*Quis inhibition of [*^*3*^*H]-L-Glu uptake after F*^*−*^* exposure*. Confluent U373 cells were pretreated with 500 µM F^−^ for 30 min, then, pre-treatment was replaced with a buffer containing 25 µM Glu, [.^3^H]- L-Glu and different Quis concentrations. Uptake was evaluated for 30 min. Results are the mean ± SEM of 3 independent experiments; each experiment was performed in quadruplicates (n = 3) and analyzed by log_10_ (inhibitor) vs response (nonlinear regression)

To characterize cystine/glutamate exchanger after 500 µM F^−^ we used sodium-free buffer and Quisqualic acid (Quis) which acts as a competitive system xc- inhibitor [54, 55]. To characterize cystine/glutamate exchanger in U373 cells, we performed a dose-dependent Quis inhibition in sodium-free conditions. [^3^H]-L-Glutamate uptake displayed an IC_50_ of 7.92 ± 1.7 µM and 5.6 ± 3.8 µM in the presence of F^−^ (Fig. 4).

**Fig. 5:**
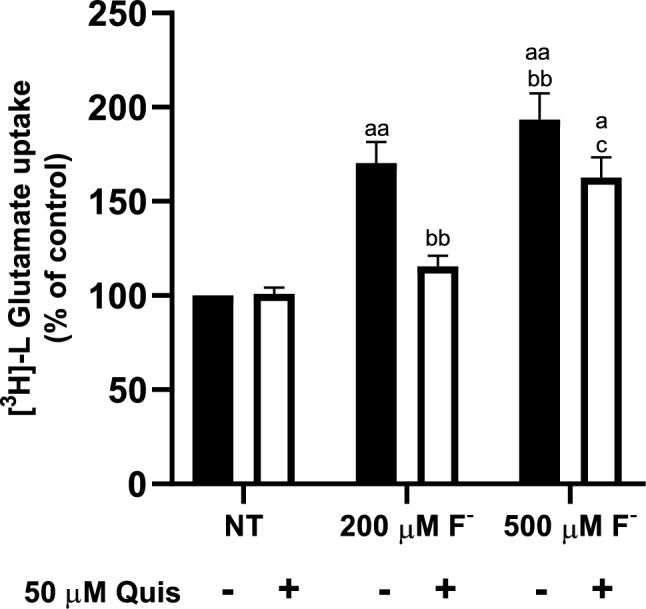
Functionality of the cystine/glutamate exchanger activity in BGC. Confluent BGC cells were pretreated with 500 µM F^−^ for 60 min, then, pre-treatment was replaced with a buffer containing 25 µM Glu, 50 µM Quis and [^3^H]-L-Glu. Uptake was evaluated for 30 min. Results are the mean ± SEM of 3 independent experiments; each experiment was performed in quadruplicates (n = 3) and analyzed by two-way ANOVA followed by Tukey multiple comparison test. ^a^p < 0.01 compared vs NT group, ^aa^p < 0.001 compared vs NT group, ^bb^p < 0.001 compared vs 200 µM F^−^, ^c^p < 0.01 compared vs 50 µM Quis + 200 µM F^−^

Previous studies have demonstrated functionality of system xc- in cerebellar BGC primary cultures and a dose-dependent Quis inhibition of [^3^H]-L-Glu uptake in Na^+^ -free conditions [31]. With this in mind, we proceed to characterize cystine/glutamate exchanger after F^−^ exposure in BGC cells, we evaluated 200 and 500 µM F^−^ exposure. As depicted in Fig. 5, a decrease in system xc- activity was detected under Quis (Fig. 5). These results and those presented in Fig. 4, fully demonstrate the expression and functionality of cystine/glutamate exchanger in U373 and BGC cultures.

### Cystine/Glutamate Exchanger Kinetic Parameters

**Fig. 6:**
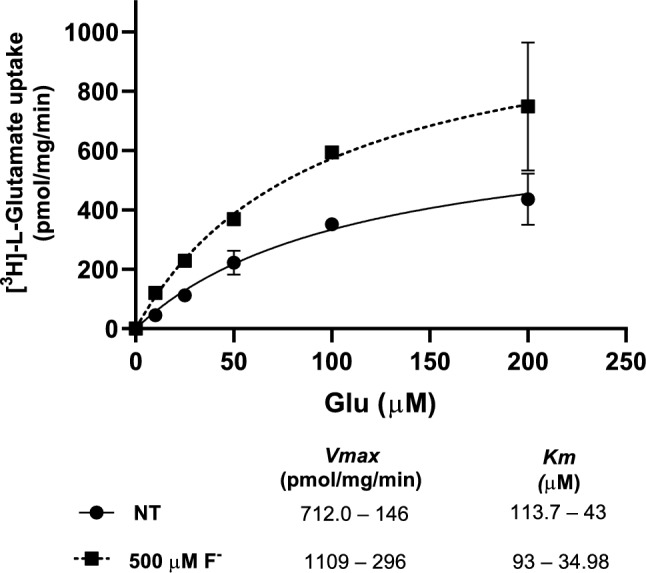
*Characterization of the kinetic parameters of [*^*3*^*H]-L-Glu uptake under F*^*−*^* exposure.* Cells were pre-treated for 30 min with F.^−^ 500 µM and then, the pre-treatment was replaced with uptake solution. Uptake was performed with 0–200 µM. To calculate the kinetic constants, curves were analyzed by Michaelis–Menten saturation assays using GraphPad Prism. Results are the mean ± SEM of 3 independent experiments each one was performed in quadruplicates (n = 3)

An increase in L-Glu uptake triggered by F^−^ exposure could be the result of an increase of functional cystine/glutamate exchanger exchangers at the plasma membrane or due to a change in the transporter catalytic rate. Therefore, we characterized the kinetic constants (K_*M*_ and V_max_) of [^3^H]-L-Glu uptake in U373-MG cells. The results shown in Fig. 6 show a 1.6-fold increase in V_max_ suggesting an increase in the number of exchangers present at the plasma membrane. The discrete change in the affinity is within the experimental error.

### Increases in System xc- Activity are Due to Increases in the Number of Exchangers at the Plasma Membrane

**Fig. 7:**
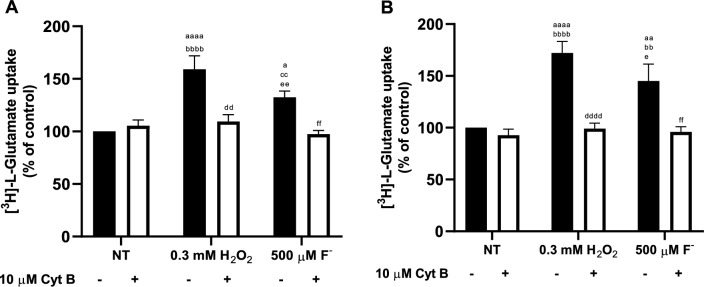
*Characterization of protein trafficking of [*^*3*^*H]-L-Glu uptake under F*^*−*^* exposure.* Cells were pre-treated for 2 h with 10 µM Cytochalasin B (Cyt B)*,* then, 500 µM F^−^ treatments were performed for **A** 30 min in U373 cells and **B** 60 min. Results are the mean ± SEM of 3 independent experiments each one was performed in quadruplicates (n = 3) and analyzed by two-way ANOVA followed by Tukey multiple comparison test. ^aa^p < 0.01 compared vs NT without Cyt B, ^aaaa^p < 0.0001 compared NT without Cyt B, ^bbbb^p < 0.0001 compared NT with Cyt B, ^cc^p < 0.01 compared NT without Cyt B, ^ddd^p < 0.001 compared con H_2_O_2_ without Cyt B, ^eeee^p < 0.0001 compared H_2_O_2_ with Cyt B, ^ee^p < 0.01 compared H_2_O_2_ with Cyt B and ^ff^p < 0.001 compared F^−^ 500 µM without Cyt B

To corroborate that the increase in exchanger activity is associated with increases in system xc- at the plasma membrane, we blocked exchanger trafficking through an inhibitor of actin polymerization, cytochalasin B (Cyt B). For this assay, a 2 h pretreatment of 10 µM Cyt B, followed by 30 and 60 min of exposure to 500 µM F^−^ for U373 (Fig. 7A) and BGC cells (Fig. 7B) respectively and 10 min of 0.3 mM H_2_O_2_ for both cellular models. Our results show an increase in activity after exposure to fluoride was decreased by blocking exchanger trafficking to the plasma membrane.

### The Antioxidant Trolox Mitigates the F^−^ Effect

**Fig. 8:**
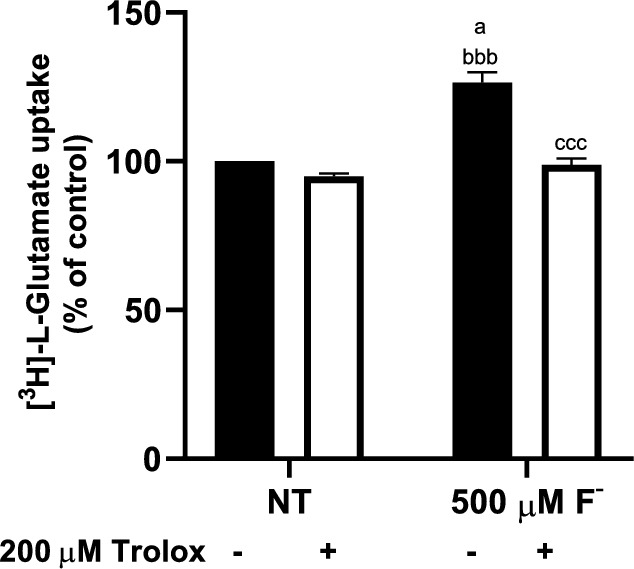
ROS *involvement in the increase in the system xc- activity mediated by F*^*−*^* exposure.* U373 cells were treated with F^−^ 500 µM for 30 min (second bar), 200 µM Trolox for 30 min (third bar), and co-exposed F^−^ and Trolox (fourth bar). Statistics analysis was performed by two-way ANOVA followed by Tukey multiple comparison test. Results are the mean ± SEM of 3 independent experiments (n = 3); each experiment was performed in quadruplicates. ^a^p < 0.01 compared vs the NT group, ^bbb^p < 0.0001 compared vs Trolox 200 µM group, ^ccc^p < 0.0001 compared vs 500 µM F^−^ group

It has traditionally been assumed that F^−^ neurotoxic effects are associated with its capacity to disrupt the redox balance, mainly through the disruption of glutathione (GSH) [56], thus altering Glu disposal that could eventually modify synaptic transmission. To investigate if the described increase in Glu uptake triggered by F^−^ could be due to an increase in ROS production, we pre-treated the cells with the antioxidant Trolox (30 min with 200 µM Trolox before F^−^ addition). Trolox mitigated the F^−^ increase of the uptake (Fig. 8), suggesting that, ROS accumulation is involved in F^−^ dependent system xc- dysregulation.

### F^−^ Modifies Cytoplasmic HuR Protein Expression

**Fig. 9:**
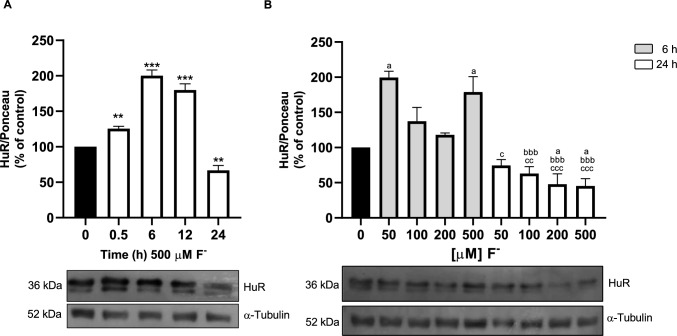
F^−^ regulates *cytoplasmatic HuR levels in U373 cells*. **A** Monolayers of U373-MG cells were treated with 500 µM F- for 0.5, 6, 12 and 24 h. **B** Cells were treated for 6 or 24 h with 50, 100, 200 and 500 µM F^−^. Cytoplasmic cell extracts were prepared and analyzed via Western blot with anti-HUR antibodies. A representative blot of four independent experiments is shown at the bottom of the graph. Graphs are the mean ± SEM of 4 independent experiments; represent the statistical analysis performed by **A** one-way ANOVA followed by Dunnett’s multiple comparison test. *p < 0.05, **p < 0.01, vs control. **B** Two-way ANOVA followed by Tukey multiple comparison test was used for panel B. ^a^p < 0.01 compared vs NT group, ^bbb^p < 0.0001 compared vs 6 h 50 µM F^−^, ^c^p < 0.01 compared vs 6 h 500 µM F^−^, ^cc^p < 0.001 compared vs 6 h 500 µM F^−^

At this point, it was evident that F^−^ augments cystine/glutamate exchanger function, and that the increase in V_*Max*_ could be interpreted as an increase in xCT expression in the plasma membrane. In this context, we decided to explore an upstream regulator of xCT. HuR, an RNA-binding protein, capable to bind to several xCT mRNA 3´-UTR regions, modifying its stability and translation [57]. Biochemical evidence of HuR expression was challenged with anti-HuR antibodies, as shown in Fig. 8, the characteristic 36 kDa is present in our cultured cells system. Interestingly, F^−^ exposure increases the amount of cytoplasmatic HuR protein in a time and concentration-dependent manner (Fig. 9A and B) pointing out an acute (as early as 30 min) as well as a long-term (6–12 h) F^−^ effect. Note that after 24 h, HuR cytoplasmic levels are decreased, which might reflect a re-shuttle to the nucleus or its degradation.

### F^−^ Increases xCT and HuR mRNA Half-Life

**Fig. 10:**
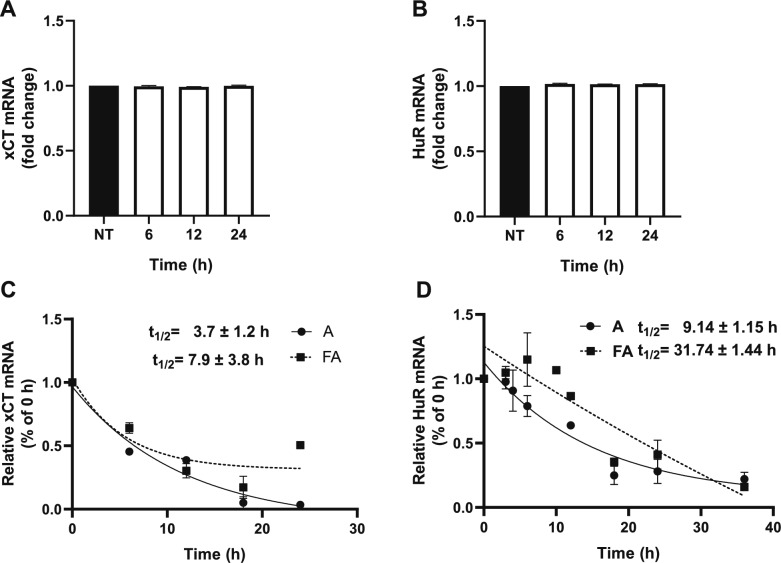
F^−^ increases *xCT and HuR mRNA stability*. Monolayers of U373-MG cells were treated with 500 µM F.^−^ for 6, 12, and 24 h. **A** xCT mRNA levels. **B** HuR mRNA levels were determined by RT-qPCR and normalized using GAPDH. **C** xCT mRNA half-life **D** HuR mRNA half-life. Transcription was stopped with 8 μM Actinomycin D (ActD)*.* For panels A and B, one-way ANOVA was followed by Dunnett’s multiple post-hoc test comparing against non-treated cells (NT). To analyze panels C and D: At the times indicated, samples were harvested for RNA isolation, and relative mRNA expression was assessed via qRT-PCR. 100% is represented as zero time. The mRNA half-life (t_1⁄2_) was calculated using nonlinear regression analysis (one-phase exponential decay) using GraphPad Prism. Graphs are the mean ± SEM of 3 independent experiments in duplicate (n = 3)

To further explore the mechanism by which F^−^ could modify the exchanger, we decided to quantify xCT and HuR mRNA levels. Surprisingly, neither xCT nor HuR mRNA levels were significantly modified (Fig. 10A and B). Taking into consideration the pivotal role of xCT in Glu extracellular regulation and according to several studies that demonstrate that the function of the exchanger and of the RNA-binding protein can be regulated at post-translational level [29, 57] we decided to explore whether the F^−^-mediated could be elicited at this level. To this end, we explored a plausible F^−^ -mediated change in xCT (Fig. 10C) and/or HuR (Fig. 10D) mRNAs half-life. An increase from 3.7 ± 1.2 to 7.9 ± 3.8 h for xCT mRNA and from 9.14 ± 1.15 h to 31.7 ± 1. 44 h for HuR mRNA is present when the cells are exposed to 500 µM F^−^.

## Discussion

F^−^ is present in natural and anthropogenic sources, however, the main source of human F^−^ exposure is drinking water [17]. Although F^−^ is recommended for dental care, chronic intake of higher than recommended concentrations results in diverse pathologies [2]. Until 2021, according to the WHO, 1.5 mg/L was set as the maximum concentration allowed for water consumption [7]. In recent years, the toxic effect of F^−^ on the CNS development has been thoroughly evaluated and the correlation between exposure and cognitive deficit is well documented [14]. Epidemiological studies carried out in regions with high F^−^ water content, have reported an increase in neurological alterations related to cognitive capacity in children such as the intelligence quotient (IQ) and disturbances of learning and memory processes [14, 58–60]. Accordingly, in vivo studies show that F^−^ can permeate the brain blood barrier (BBB) after a chronic exposure [61, 62]. This ion can be found in the hippocampus, cerebral cortex and cerebellum, regions known to be involved in learning and memory [3, 10, 14].

Although it is not clear whether neurons or astrocytes are more susceptible to F^−^ cellular stress, it is known that astrocytes outnumber neurons in most brain areas and that these cells are critically involved in energy support for neurons [63]. Glial functions are critical for neuron maintenance and protection, particularly under acute or chronic injury [64]. Moreover, neurons are highly susceptible to cellular stress [65], demanding a constant GSH supply, favoring an increased glial Cys uptake through the cystine/glutamate exchanger.

As a first approach to gain a deeper insight into F^−^ brain deleterious effects, we analyzed the toxicity of different F^−^ concentrations (10 to 1000 µM) on two glial models: U373-MG cells and chick cerebellar BGC. The rationale for this choice is simple, glioma cells are known to overexpress the exchanger, whereas Bergmann glia cells are roughly half of the cerebellar cells, structure known to accumulate F^−^. We did not detect a significant effect in the cell viability in both cellular systems [49]. Taking into consideration that glial cells outnumber neurons and that an exquisite glial-neuronal interplay takes place in glutamatergic synapses highly enriched in F^−^ accumulated structures like the cerebellum and the hippocampus we decided to use two astrocytic models, one with a recognized increase in the exchanger expression (U373-MG cells) and one extremely characterized in terms of its involvement in Glu recycling (BGC) and that is also involved in Glu-dependent gene expression regulation [66]. As the most electronegative element, F^−^ effects on cellular components are very extensive, it can disrupt signaling cascades and other regulatory processes such as post-translational regulators [58]. We focused on glutamatergic transmission; Glu has a pivotal role in synaptic plasticity, and its molecular regulation correlates with neurodevelopment [67]. Within the synaptic cleft, Glu levels are mainly regulated by the Glu/Gln shuttle clearing extracellular Glu through different sodium-dependent glial Glu transporters (EAAT1 and 2) and the cystine/glutamate exchanger [68].

Using chick cerebellar BGCs, we have documented that radial glia cells are sensitive to F^−^. An increase in Glu uptake mediated by EAAT1, is present and a transient decrease in polypeptide elongation is evident after F^−^ treatment [49]. In the human retinal MIO-M1 Müller glia cells, F^−^ exposure disrupts Gln transport resulting in a deficient Glu turnover [15]. In order to widen our knowledge of F^−^ brain effects and support the notion of glia cells as a target for toxicants and by these means, disruptor of glutamatergic transmission, we focused here on a plausible modification of the cystine/glutamate exchanger, given its role in excitotoxicity [27]. We could demonstrate here that F^−^ exerts a biphasic effect on xCT: an acute effect in its function, most possible related to its plasma membrane levels that might be sustained in longer time periods through a stabilization of its mRNA.

HuR is predominantly located within the nucleus in control conditions, however, HuR can be translocated to the cytosol and bind to different mRNAs when stimulated by exogenous agents such as stressors [57]. As our first approach, we decided to evaluate whether HuR protein was modified after exposure to F^−^, for this purpose, we performed cytoplasmic protein extracts, and our data indicate that after 6 h treatment, HuR has its maximum cytoplasmatic increase (Fig. 9). Although a Western blot approach would be ideal to quantify xCT protein levels after F^−^ exposure, no commercially available antibodies can identify the characteristic 56 kDa band reported by Ottestad-Hansen with a verified antisera challenged with xCT knock out mice, compromising the use of the mentioned reagents [25]. In this scenario, we focused on measuring xCT mRNA levels. To our surprise, no changes in its mRNA levels were found upon F^−^. A similar response (no change) was found when one of its upstream regulators, HuR, mRNA levels were measured after F^−^ exposure. To better understand the mechanism by which xCT could be upregulated, and taking into consideration the xCT mRNA harbors more than 10 ARE sequences within its 3'-UTR (see NCBI Reference Sequence: NM_014331.4), we measured xCT and HuR mRNAs half-life [69]. Our results suggest that F^−^ treatment leads to a stabilization of HuR and xCT mRNAs favoring xCT mRNA translation (Fig. 10). The involvement of HuR is an attractive explanation but needs further experimentation that is clearly out of the scope of this contribution. In any event the fact that HuR is also altered by F^−^ is itself an important piece of information into the molecular mechanisms of toxicity of this halogen. It is important to mention here that in silico and immunoprecipitation studies demonstrate a HuR- xCT mRNA binding after interleukin 1β (IL- 1β) treatment in primary mouse astrocytes [27].

Taking into consideration that the cystine/glutamate exchanger is present in the astrocytic end feet [29], this exchanger is mainly involved in the maintenance of extra-synaptic Glu levels. Augmentation of Glu release mediated by this exchanger could activate extra-synaptic NMDA2B receptors associated with excitotoxic insults [70]. In this context, it should also be noted that xCT overexpression is associated to cognitive deficits [71, 72] supporting the idea of a plausible connection between these two events.

**Fig. 11:**
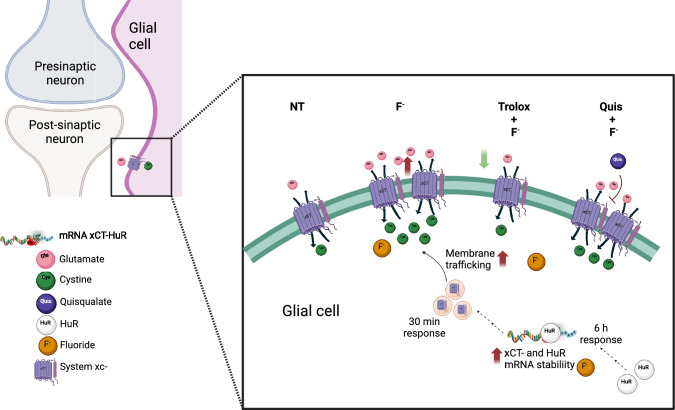
Summary *of our current findings, depicting the effect of F*^*−*^* exposure on glutamate transporters, increasing system xc- activity.* Representation of a plausible F^−^ cascade of events. Created by Bioorender.com agreement number HY256GHDSS

F^−^ increases intracellular calcium concentrations as well as decreases the activity of Ca^2+^ ATPase and leads to hypercalcemia [73]. It is important to note that the regulation of HuR stability is a complex process influenced by post-translational modifications, such as PKCα and PKCδ serine phosphorylation [74]. Taking into consideration that radial glia Glu receptors activate PKC [75] it is tempting to speculate that Glu itself regulates its transport and that this regulation loop is a target of F^−^ toxicity. The cystine/glutamate exchanger is constitutively expressed in astrocytes; however, stressful stimuli, like F^−^_,_ can lead to its overexpression. It should be noted that proteins are dynamically regulated through biochemical transactions that might occur simultaneously: a rapid increase in plasma membrane exchangers via the regulation of its turnover (trafficking) and a slower response that is most possible through translational control by epigenetic mechanisms such as mRNA stability. A summary of our findings is shown on Fig. 11.

## Conclusion

Our data supports the idea of a critical involvement of glial cells in F^−^ toxicity. Work currently under progress in our group is aimed at the characterization of the signaling pathways that regulate HuR function under F^−^ exposure as a preliminary step to further understand the complexity of the molecular mechanisms of F^−^ toxicity.

## Data Availability

No datasets were generated or analysed during the current study.
